# Dietary Nitrate: Where Is the Risk?

**DOI:** 10.1289/ehp.114-1552029

**Published:** 2006-08

**Authors:** Jean-Louis L’hirondel, Alex A. Avery, Tom Addiscott

**Affiliations:** Service de Rhumatologie, Centre Hospitalier Universitaire de Caen, Caen, France, E-mail: lhirondel-jl@chu-caen.fr; Hudson Institute, Center for Global Food Issues, Churchville, Virginia, E-mail: aavery@rica.net; Rothamsted Research, Harpenden, Herts, United Kingdom, E-mail: tom.addiscott@ukf.net

Links between nitrate and health risk have been studied for > 50 years, resulting in a large body of research. As two book-length reviews of the issue (Addiscott 2005; L’hirondel and L’hirondel 2001) tried to show, none of the health claims against dietary nitrate have been substantiated.

If there was not already an established maximum contaminant level (MCL) of 10 ppm for nitrate in drinking water in the United States [U.S. Environmental Protection Agency (EPA) 1991; World Health Organization (WHO) 1958], it would be extremely difficult, if not impossible, to justify one based on the extensive evidence gathered to date.

The conclusion of Ward et al. (2005) in their article “Drinking-water Nitrate and Health—Recent Findings and Research Needs” (Ward et al. 2005) is somewhat different. Although they were not able to reasonably conclude that dietary nitrates elevate a single health risk, the authors insisted that possible risks “must be more thoroughly explored before changes to nitrate water quality standards are considered.” Why?

Examining methemoglobinemia, cancer, reproductive, and other potential risks, Ward et al. (2005) presented the extensive body of research demonstrating only very slight negative, very slight positive, or no correlation (and usually all three). This is exactly what one would expect if there were no actual correlation. Yet instead of reexamining the MCL, Ward et al. recommended further searching for a justification of the 50-year-old regulation (WHO 1958).

We would like to draw attention to a few key additional points.

First, although the U.S. MCL for drinking water nitrate is 45 mg/L (U.S. EPA 1991), nitrate concentrations in vegetables may be > 50 times higher; vegetables often contain > 2,000–3,000 mg nitrate per kilogram. Yet nitrate-rich vegetables are good for health.

Ward et al. (2005) seem aware of this point, as they stated that “intake of dietary nitrate is less likely to increase nitrosation, because of the presence of nitrosation inhibitors in vegetables.” However, they forgot the metabolism of nitrate in humans. Salivary nitrate (not dietary nitrate) is reduced to nitrite in the mouth. In fact, plasma nitrate is extracted by the salivary glands and secreted at high concentrations in saliva; in adults and children > 6 months of age, a fraction of this salivary nitrate is converted in the mouth to nitrite. Nitrite levels in saliva are maximal 20–60 min after nitrate intake. Also, because of the acidity of the gastric juice (Dang Vu et al. 1994), nitrite concentrations in gastric juice are extremely low; 15-fold to several hundredfold less than that of salivary nitrite.

Regarding the cancer risks of nitrate, if drinking water with 10–20 ppm nitrate-nitrogen (nitrate-N) were toxic, vegetables (with their comparatively high nitrate levels) would likely also be toxic, in spite of the presence of reputed nitrosation inhibitors.

Second, during the last 12 years, several works have indicated beneficial effects of nitrate due to its conversion in the body into nitrite (NO^2−^), nitric oxide, and diverse reactive compounds. The studies carried out since 1994 by the teams of Benjamin and Duncan are worth noting (Benjamin 2000; McKnight et al. 1999). Also, a meeting was held in Bethesda, Maryland, 8–9 September 2005 under the aegis of the National Institutes of Health and devoted to the “Role of Nitrite in Physiology, Pathophysiology and Therapeutics” (Gladwin et al. 2006).

The current MCL for nitrates of the United States, Europe, and World Health Organization are all based on the flawed American Public Health Association survey from 1948 in which “special emphasis was placed on restricting the data to those [infantile methemoglobinemia cases] definitely associated with nitrate-contaminated water” (Walton 1951). This requirement ensured the inclusion of any suspected (although not proven) case of infantile methemoglobinemia where nitrate levels were even slightly above background (~2–5 ppm). A mere five suspected cases in that survey were reported at 10–20 ppm nitrate-N , and in some cases the water was tested months after the cyanotic episode.

The societal costs of complying with the current MCL are growing, especially in rural communities least economically capable of shouldering the high costs per person of nitrate-ion removal. This economic burden imposed with questionable medical basis seems to have completely escaped Ward et al. (2005).

Although we are not against continued study to ensure adequate protection of public health, it seems to us that more than enough evidence has been gathered to confidently say that nitrates are not the threat they were once thought to be. Raising the drinking water MCL for nitrates to 20 ppm nitrate-N to reflect the extensive body of research would relieve many small rural communities of a significant economic burden without adding appreciably to any known health risks.
